# Collagen XVII: A Shared Antigen in Neurodermatological Interactions?

**DOI:** 10.1155/2013/240570

**Published:** 2013-06-26

**Authors:** Allan Seppänen

**Affiliations:** Vanha Vaasa Hospital, 65380 Vaasa, Finland

## Abstract

Collagen XVII is a nonfibril-forming transmembrane collagen, which functions as both a matrix protein and a cell-surface receptor. It is particularly copious in the skin, where it is known to be a structural component of hemidesmosomes. In addition, collagen XVII has been found to be present in the central nervous system, thus offering an explanation for the statistical association between bullous pemphigoid, in which autoimmunity is directed against dermal collagen XVII, and neurological diseases. In support of the hypothesis that collagen XVII serves as a shared antigen mediating an immune response between skin and brain, research on animal and human tissue, as well as numerous epidemiological and case studies, is presented.

## 1. Introduction

Collagens, the most abundant protein in the human body [1], are a family of extracellular or transmembrane proteins defined by a triple helical structure, which is formed by hydrogen bonds between three polypeptide strands called *α*-chains [2]. Collagens have previously been overlooked for roles in the brain, since fibrillar collagens, the best known subfamily of collagens, are not present in the mature central nervous system (CNS), except in marginal structures such as the meninges [3] and the basement membrane in the blood-brain barrier [4]. However, gradually accumulated evidence has made it apparent that collagens are not merely structural proteins giving strength to tissue, but bioactive molecules with a dynamic role within the nervous system [5, 6]. In fact, a role in the CNS, albeit often transient, has now been identified for nearly every type of collagen during some phase of CNS development. Decisive functions, such as establishment of brain architecture [7], neuronal differentiation [8], regulation of axonal outgrowth [9] and targeting [10], and synaptic differentiation [11], have been attributed to different collagens during various stages of neural maturation [5, 12]. 

## 2. Molecular Structure and Expression of Collagen XVII

Collagen XVII, also known as bullous pemphigoid antigen 2 (BPAG2) or BP180, due to its 180 kDa mass, belongs to the subfamily of non-fibril-forming transmembrane collagens, all four members of which function as both matrix proteins and cell-surface receptors. These proteins exist in two different forms, one being a type II-oriented transmembrane protein and the other being a shorter soluble molecule derived by posttranslational proteolysis [13, 14]. 

The structure of collagen XVII and its binding ligands have been previously described in detail [14, 15]. It is a homotrimer of three 180 kDa *α*-chains, each with a long intracellular N-terminal domain, a short transmembrane stretch, and an extracellular C-terminus [16]. Collagen XVII is known to be a structural component of hemidesmosomes, which mediate adhesion of epidermal keratinocytes and certain other epithelial cells to the underlying basement membrane. The intracellular component of collagen XVII interacts with the *β*4-integrin subunit, plectin, and BPAG1 [17, 18] to form a stable attachment of hemidesmosomes to keratin intermediate filaments within the cell. The 120-kDa ectodomain of collagen XVII binds to both the *α*6 integrin subunit [19] and laminin 332 and is shed from the cell surface by the metalloproteases ADAM 9 and ADAM 10 [20], yielding a soluble form of the molecule into the extracellular matrix [21, 22]. Although the physiological implications of the shedding are not certain, it has been proposed that this allows the anchored cell to detach, migrate, and differentiate during morphogenesis and during regeneration in wound healing [15, 23]. 

In the nervous system, collagen XVII has been studied in both animal and human studies. In bovine and rat tissue it has been detected, often colocalizing with its epithelial ligand BPAG1 and complexing with various laminins, in Muller glial cells, photoreceptors and synaptic regions of the retina, and the cerebellum [24]. In human brain tissue, collagen XVII has been shown to localize predominantly to the soma and proximal axons of neurons, the level of expression varying from one brain structure to another; areas with a strong presence of collagen XVII are the hypoglossal nucleus (nucleus XII), oculomotor nucleus (nucleus III), nucleus basalis of Meynert, supraoptic nucleus, subthalamic nuclei, and pyramidal cells of the hippocampal regions CA 4-2 and the ganglionic layer of the cerebral cortex, particularly Betz cells [25, 26]. On an intracellular level, collagen XVII seems to be concentrated in irregularly-shaped autofluorescent granules around the nucleus, which, using electron microscopy, have been confirmed to be lipofuscin [27]. 

## 3. Collagen XVII, Bullous Pemphigoid, and Neurological Disease

Lack of collagen XVII or the loss of its function results in diminished epidermal adhesion and skin blistering in response to minimal shearing forces. In non-Herlitz-type junctional epidermolysis bullosa this can be caused by mutations in the gene coding collagen XVII, COL17A1 [28]; in the pemphigoids, that is, bullous pemphigoid (BP), pemphigoid gestationis, linear igA disease and mucous membrane pemphigoid, the cause of epidermal impairment is autoimmunity against collagen XVII [29–31]. The autoantibodies are primarily directed against the immunodominant N-terminal of the juxtamembranous NC16a domain [32], which is one of the noncollagenous sequences of collagen XVII's extracellular part [33]. However, collagen XVII's hemidesmosomal ligand BPAG1 can also serve as an autoantigen in BP [34], which is the most frequent pemphigoid; it has an annual incidence varying in different studies from 2.6 to 42.8 per million population [35]. It usually affects the elderly, and both genders are similarly affected. Clinically, it is characterized by tense blisters, variably associated with severe itching. 

Interestingly, robust statistical associations between BP and neurological disorders have been repeatedly published, particularly in relation to dementia and cerebrovascular disease (Table 1). Foureur et al. [36] found neurological disorders in 65% (*n* = 30/46) of consecutive day-unit patients with BP, chief amongst which were senile dementia and cerebral stroke. Likewise, Cordel et al. [37] found that 36% (*n* = 123/341) of BP patients from French dermatology departments had a neurological disorder: 55% of these were dementia, primarily Alzheimer's disease followed by vascular dementia, and 42% were cerebral stroke. These findings were repeated by Jedlickova et al. [38]. Their analysis showed that psychoneurological disease, again primarily cerebral stroke and dementia, was found in 42.7% of 89 BP patients but only in 19.1% of controls. Similar figures were reported by Taghipour et al. [39]: at least one neurological disease was present in 46% of 90 consecutive BP patients from an immunobullous referral centre, as compared to 11% in controls. Identically, a significant association with BP was found for cerebrovascular disease and dementia. Dementia, or severe cognitive impairment, has since been consistently reported in association with BP in three more studies [40–42] and a case report [43]. What is more, a study of 138 elderly subjects with no dermatological symptoms showed that the presence of anticollagen XVII antibodies in the serum was significantly correlated with a mini-mental test score of under 24/30, that is, the cut-off score for dementia [44].

Also, multiple sclerosis and Parkinson's disease have been associated with bullous pemphigoid. A retrospective study of the discharge records of all hospitalized patients in a region in northern Italy supported an association between bullous pemphigoid, multiple sclerosis, and Parkinson's disease [45]. In line with these findings, the literature reports several cases of bullous pemphigoid developing in patients with multiple sclerosis [46–48] and at least one in a patient with Parkinson's disease [49]. The association of Parkinson's disease with bullous pemphigoid has been subsequently reported in epidemiological studies others by as well [37, 40–42]. There are also case reports of unilateral BP on the paralyzed side of hemiplegic patients [36, 50, 51]. 

Psychiatric morbidity may also be associated with BP [52]. Bastuji-Garin found that unipolar or bipolar mood disorders and the use of psycholeptics, particularly neuroleptics, were a risk factor for BP in the elderly [41, 53]. One large study has also associated schizophrenia with BP in females [42].

Although BP usually affects people over 65 years of age, cases among younger people are not unheard of. Interestingly, a retrospective study of 74 BP patients under the age of 60 found neurological disorders in 12 and use of psychiatric drugs in 33 cases [54] (not included in Table 1 due to age difference of cases as compared to the other studies).

In light of the association between BP and neurological impairment, it is notable that neurological symptoms are not associated with junctional epidermolysis bullosa [55]. This suggests that collagen XVII impairment in itself does not explain the neurodermatological symptoms but, rather, the pathological autoinflammatory reaction against collagen XVII, feasibly by increasing vascular permeability and favouring transendothelial migration of inflammatory cells. In fact, it has recently been revealed that collagen XVII itself has an important role in regulating inflammatory chemotaxis: depending on the type of inflammatory stimuli, the expression level of collagen XVII can have either an enhancing or suppressing effect on IL-8, a proinflammatory chemokine [56]. 

## 4. The Possible Role of Collagen XVII as a Common Antigen in Neurological and Immunobullous Skin Disorders

The fact that collagen XVII is present in human brain tissue raises the possibility of collagen XVII having a role in neurological disorders, particularly in association with subsequent BP. Interestingly, BPAG1, the other antigen targeted in BP, also has variants that are expressed in the nervous system [57–59] and could thus also serve as an autoantigen in the BP-associated neurological disorders. Indeed, antibodies in the serum of patients with both BP and various neurological diseases have been shown to recognize both a 180 kDa and a 230 kDa protein corresponding to collagen XVII and BPAG1, respectively, in mouse and human brain extract [57, 60] and human epidermal extract [61, 62]. What the relative significance of these two antigens is in neurological disease is unclear: previous studies have revealed only a low frequency of reactivity in the immunoblotting of the cerebrospinal fluid of MS patients (without BP) against BPAG1 [58, 62]. Also, in a study of collagen XVII and BPAG1 autoantibodies in the serum of 337 individuals with no signs of BP [63], of the 25 found to be positive, 5 had neurologic diseases listed in their medical records; in all but one case the amount of collagen XVII antibodies was greater than the amount for BPAG1 (correspondence [64]). On the other hand, Soni et al. [65] found evidence of neither collagen XVII nor BPAG1 antibodies in a case of autoimmune encephalopathy with BP.

In any case, when both BP and neurological disorder are present, neurological disease usually precedes BP [40, 60] by months to years [37, 45]. This supports the idea that neuronal antigen exposure, conceivably via a compromised blood-brain barrier, is causatively involved in subsequent development of BP. In light of this, the hypothesis of immunoglobulin-mediated neurodegeneration in Alzheimer's disease [66] is intriguing, as dementia is the disorder most consistently associated with BP. This hypothesis involves an age-related compromise of the blood-brain barrier [67] and loss of the “immunological privilege” of the brain. In a murine model of senescence, vascular permeability to the brain has been shown to be particularly prevalent in the hippocampal region [68], where the present author et al. have shown strong collagen XVII expression [25, 26], and which is a well-recognized predilection area for Alzheimer's disease- (AD-)related lesions [69]. It is also interesting that our previous results, albeit based on samples from a single brain (male, 68 years of age at death), show that collagen XVII expression was confined to lipofuscin [27], as oxidative stress is intimately associated with the aging process and AD [70]. Lipofuscin is primarily composed of nondegradable, oxidatively modified macromolecule residues which largely originate from autophagocytosed mitochondria [71], the organelle most affected by the oxidative damage caused by free radicals emanating from the respiratory processes [72]. Lipofuscin has usually been considered waste material, the accumulation of which has a detrimental effect on various cellular functions [73], although this view has been questioned as the variability of lipopigments and sytosolic degradation pathways has been expounded [74]. It has also been suggested that lipofuscin actually benefits the neuron by incorporating potentially damaging metabolites [75]. However, it is worth considering that in a younger brain the intracellular location of collagen XVII could be different, as lipofuscin accumulates in the aging neuron; thus the findings concerning the intraneuronal localization of collagen XVII must be viewed as preliminary.

The other condition most frequently reported in association with BP is cerebrovascular disease, or stroke (Table 1). Of course, etiopathogenetically, this is not a neuronal disease but vascular in origin. Therefore, it is interesting that in contrast with neurological disease in general, stroke has been shown to often occur after the onset of BP [76]. This could be explained by the fact that the inflammatory state present in BP is not confined to the skin but also involves vascular endothelium [77], as does the pathogenesis of atherosclerosis, atheroma plaque rupture, and thrombosis [78]. Also, the inflammatory response in BP involves raised levels of eosinophils not only in the skin lesions but also in peripheral blood [79] and it has been shown that eosinophil granulocytes are an important source of tissue factor, the initiator of blood coagulation, in human blood [80, 81]. In addition, there is some evidence that antiphospholipid antibodies are present in the serum of BP patients [82]. Thus, the BP-associated inflammation, together with the hypercoagulable state associated with eosinophilia and antiphospholipid antibodies, could exacerbate preexisting atherosclerosis and promote thrombosis and stroke [76].

In terms of inflammation, the findings linking affective disorder and schizophrenia with BP [41, 42] are interesting, as there is evidence of upregulation of immune response genes in these disorders [83]. In fact, for schizophrenia, a neuroimmune hypothesis has been debated for decades [84], most recently in terms of NMDA-receptor autoimmunity [85, 86]. Although the current consensus concerning the inflammatory etiology of schizophrenia involves the idea of a long-lasting consequence of an infective-immune challenge during early brain development, numerous other explanations have been offered, including autoimmunity towards certain brain structures [87], particularly in the hippocampus [88]. However, as inflammation is also closely linked with behavioral parameters such as exercise, alcohol abuse, and smoking, as well as with medical conditions including coronary artery disease, obesity, and insulin resistance [89], interpreting the inflammatory findings in psychiatric disease is exceedingly complex. Added to this is the fact that collagen XVII itself has now been shown to have a dynamic role in inflammatory responsivity [56]; whether this can in any way be linked to BP-associated neuropsychiatric morbidity remains to be seen. 

All in all, the findings presented previously support the idea of collagen XVII being a worthwhile focus of study in the area of neuroimmunology. Although the statistical studies linking BP with neurological disorders are robust, further biological studies are needed to answer questions relating to both the physiological and pathogenetic roles of collagen XVII in the CNS. 

## Figures and Tables

**Table 1:** Epidemiological studies linking bullous pemphigoid and neurological morbidity.

Reference	Country of study	Type of study	Number of BP cases in study	Neurological disorders associated to BP
Foureur et al., 2001 [36]	France	Retrospective case control	46	Senile dementia/Alzheimer Cerebral stroke

Stinco et al., 2005 [45]	Italy	Retrospective	238	Multiple sclerosis Parkinson's disease

Cordel et al., 2007 [37]	France	Retrospective	341	Dementia Cerebral stroke Parkinson's disease or parkinsonism

Jedlickova et al., 2010 [38]	Czech Republic	Retrospective case control	89	Dementia Cerebral stroke

Taghipour et al., 2010 [39]	UK	Retrospective case control	90	Cerebrovascular disease Dementia

Langan et al., 2011 [40]	UK	Retrospective population based case-control	868	Dementia Parkinson's disease Stroke Epilepsy

Bastuji-Garin et al., 2011 [41]	France	Prospective case control	201	Severe cognitive impairment (MMSE < 17) Parkinsons' disease Uni- or bipolar disorder Long-term neuroleptic drug use

Chen et al., 2011 [42]	Taiwan	Retrospective population based case control	3485	Dementia Stroke Schizophrenia Epilepsy Parkinson's disease
